# Modulation of Endothelial Inflammation by Low and High Magnitude Cyclic Stretch

**DOI:** 10.1371/journal.pone.0153387

**Published:** 2016-04-29

**Authors:** Yufeng Tian, Grzegorz Gawlak, James J. O'Donnell, Isa Mambetsariev, Anna A. Birukova

**Affiliations:** Lung Injury Center, Section of Pulmonary and Critical Medicine, Department of Medicine, University of Chicago, Chicago, Illinois 60637, United States of America; University of Illinois College of Medicine, UNITED STATES

## Abstract

Excessive mechanical ventilation exerts pathologic mechanical strain on lung vascular endothelium and promotes endothelial cell (EC) inflammatory activation; however, the specific mechanisms underlying EC inflammatory response caused by mechanical ventilation related cyclic stretch (CS) remain unclear. This study investigated the effects of chronic exposure to CS at physiologic (5%) and pathologic (18%) magnitude on pulmonary EC inflammatory status in control conditions and bacterial lipopolysacharide (LPS)-stimulated conditions. EC exposure to high or low CS magnitudes for 28–72 hrs had distinct effects on EC inflammatory activation. 18% CS increased surface expression of endothelial adhesion molecule ICAM1 and release of its soluble form (sICAM1) and inflammatory cytokine IL-8 by CS-stimulated pulmonary endothelial cells (EC). EC inflammatory activation was not observed in EC exposed to 5% CS. Chronic exposure to 18% CS, but not to 5% CS, augmented ICAM1 and IL-8 production and EC monolayer barrier disruption induced by LPS. 18% CS, but not 5% CS, stimulated expression of RhoA GTPase-specific guanine nucleotide exchange factor GEF-H1. GEF-H1 knockdown using gene-specific siRNA abolished 18% CS-induced ICAM1 expression and sICAM1 and IL-8 release by EC. GEF-H1 knockdown also prevented disruption of EC monolayer integrity and attenuated sICAM1 and IL-8 release in the two-hit model of EC barrier dysfunction caused by combined stimulation with 18% CS and LPS. These data demonstrate that exacerbation of inflammatory response by pulmonary endothelium exposed to excessive mechanical stretch is mediated by CS-induced induction of Rho activating protein GEF-H1.

## Introduction

Ventilation support of critically ill patients with or without pre-existing lung pathology exerts excessive mechanical strain on certain regions in the lung. This excessive mechanostimulation compromises the blood-gas barrier, and increase lung vascular permeability, which may ultimately lead to massive vascular barrier dysfunction, pulmonary edema and ventilator-induced lung injury (VILI) [1, 2]. Lung vascular barrier dysfunction in VILI conditions is also accompanied by increased production of inflammatory cytokines such as TNFα, IL-8, and IL-1 [1, 3–5].

Exposure of lung cells to excessive mechanical stretch, which is caused by suboptimal mechanical ventilation, and pro-inflammatory bacterial compounds present in clinical settings synergistically contribute to the severity of lung injury and acute respiratory distress syndrome (ARDS). The “two-hit” model of acute lung injury wherein endotoxin exposure is combined with mechanical ventilation represents a clinical scenario in ALI/ARDS patients and remains an area of active research [6]. Clinical data and experimental observations indicate that besides direct effects on epithelial and endothelial integrity and permeability [7, 8], mechanical ventilation at high tidal volumes causes release of inflammatory cytokines which further exacerbate ventilator-induced lung injury [9, 10]. Mechanical ventilation at high tidal volumes has been also shown to enhance LPS-induced lung injury and vascular leak in animal models [11].

At the molecular level, the signaling cross-talk behind the synergistic effects of pathologically relevant levels of CS, vasoactive agonists, or inflammatory agents involve activation of Rho GTPase, Rho-associated kinase, and myosin light chain phosphorylation [12, 13]. High magnitude CS promotes Rho activation related to the early phase of thrombin-induced EC monolayer disruption, and suppresses Rac activation essential for the recovery phase [12, 14]. In comparison to 18% CS, physiological CS causes lower levels of thrombin-induced Rho activation, reduces EC barrier disruption and significantly promotes the EC monolayer recovery phase associated with increased Rac GTPase activities [12–14].

Guanine nucleotide exchange factor H1 (GEF-H1) is a Rho-specific GEF [15]. GEF-H1 localization on microtubules suppresses its guanine-exchange activity, whereas GEF-H1 release from microtubules and targeting to focal adhesion complexes induced by mechanical forces stimulates GEF-H1 nucleotide exchange activity and activation of the Rho pathway [16, 17]. Recent studies also show GEF-H1’s role in endothelial response to inflammatory stimulation [18–20]. In addition to rapid Rho activation and exacerbation of agonist-induced permeability in lungs and cells subjected to acute high magnitude mechanical strain, chronic CS preconditioning additionally regulates agonist-induced EC barrier regulation by transcriptional mechanisms [21–24]. This study examined the effects of chronic exposure to CS at physiologic (5%) and pathologic (18%) magnitude on pulmonary EC inflammatory activation and investigated the role of GEF-H1 expression in the mediation of chronic CS effects.

## Materials and Methods

### Cell culture and reagents

Human pulmonary artery endothelial cells were obtained from Lonza (Allendale, NJ). Cells were maintained according to the manufacturer’s recommendations and used for experiments at passages 5–7. Bacterial lipopolysaccharide (LPS) (Escherichia coli O127:B8) was obtained from Sigma-Aldrich (St. Louis, MO). Antibodies to ICAM1 were obtained from Santa Cruz Biotechnology (Santa Cruz, CA); antibodies to GEF-H1 were from Cell Signaling (Beverly, MA). All reagents for immunofluorescence were purchased from Molecular Probes (Eugene, OR). Unless specified, reagents were obtained from Sigma (St. Louis, MO).

### Cell culture under cyclic stretch

Cyclic stretch (CS) experiments were performed as previously described [21, 25] using FX-4000T Flexecell Tension Plus system (Flexcell International, McKeesport, PA) equipped with a 25 mm BioFlex Loading station designed to provide uniform radial and circumferential strain across a membrane surface along all radii. Each BioFlex membrane is stretched over the post when under vacuum pressure, creating a single-plane uniformly stretched circle. The radial and circumferential strain were experimentally determined by the vendor (Flexcell International). The cells were exposed to high magnitude (18% linear elongation, sinusoidal wave, 25 cycles/min) or physiologic (5% linear elongation) cyclic stretch for 48–72 hrs to recapitulate the mechanical stresses experienced by the alveolar endothelium at high tidal volume mechanical ventilation [21, 26]. Control BioFlex plates with static EC culture were placed in the same cell culture incubator and processed similarly to CS-preconditioned cells. Lastly, cell lysates were collected for western blot analysis, or CS-exposed endothelial monolayers were fixed and used for immunofluorescence staining.

### Knockdown of GEF-H1 in pulmonary EC

To reduce the content of endogenous GEF-H1 cells were treated with gene-specific siRNA duplexes. Pre-designed standard purity siRNA sets (*Homo sapiens*) were ordered from Dharmacon (Lafayette, CO), and transfection of EC with siRNA was performed as previously described [27, 28]. After 24 hrs of transfection cells were used for chronic CS experiments. Western blot verification of specific protein depletion was performed at the end of CS experiments. Nonspecific, non-targeting siRNA (Dharmacon, Lafayette, CO) was used as a control treatment.

### Expression plasmids and transient transfection protocol

Plasmids encoding wild type GEF-H1 (1–894) and dominant negative mutant, GEF-H1-DN bearing EGFP-tag have been previously described [16]. Transient transfections of human pulmonary EC with GEF-H1 plasmids were performed as described by our group [17, 29].

### Western blot

Confluent HPAEC exposed to 18% CS or static conditions were treated with vehicle or LPS. Protein extracts from EC homogenates were separated by SDS-PAGE, transferred to polyvinylidene difluoride membranes, and the membranes were incubated with specific antibodies of interest. Equal protein loading was verified by reprobing membranes with β-actin antibodies. Immunoreactive proteins were detected with the enhanced chemiluminescence detection system according to the manufacturer’s protocol (Amersham, Little Chalfont, UK).

### Immunofluorescence

Endothelial monolayers grown on BioFlex plates were exposed to cyclic stretch and subjected to immunofluorescence staining as described previously [17]. Membranes with attached cells were mounted on 4x4 cm rectangular coverslips and analyzed using a Nikon video imaging system (Nikon Instech Co., Tokyo, Japan). Images were processed with Image J software (National Institute of Health, Washington, USA) and Adobe Photoshop 7.0 (Adobe Systems, San Jose, CA) software. Quantitative analysis of paracellular gap formation was performed as previously described [21, 30, 31]. The gap formation was expressed as a ratio of the gap area to the area of the whole image; the values were statistically processed using Sigma Plot 7.1 (SPSS Science, Chicago, IL) software. For each experimental condition at least 10 microscopic fields in each independent experiment were analyzed.

### Measurement of IL-8, TNFα, IL-6 and soluble ICAM1

For IL-8, TNFα, IL-6 cytokines and soluble ICAM1 (s-ICAM1) measurements, preconditioned medium of human pulmonary EC cultures exposed to cyclic stretch or static conditions was collected and centrifuged to remove debris. Cytokine and s-ICAM1 levels were determined by ELISA (R&D Systems, Minneapolis, MN) following manufacturer’s protocol. Absorbance was recorded at 450 nm in microplate reader (Thermomax; Molecular Devices, Menlo Park, CA).

### Statistical analysis

Results are presented as mean ± SD. Stimulated samples were compared to controls by unpaired Student’s t-test. For multiple-group comparisons, a one-way analysis of variance (ANOVA), followed by the post hoc Tukey test, were used. P<0.05 was considered statistically significant.

## Results

### Differential effects of chronic CS at high and low magnitudes on pulmonary EC inflammatory activation

**Fig 1:**
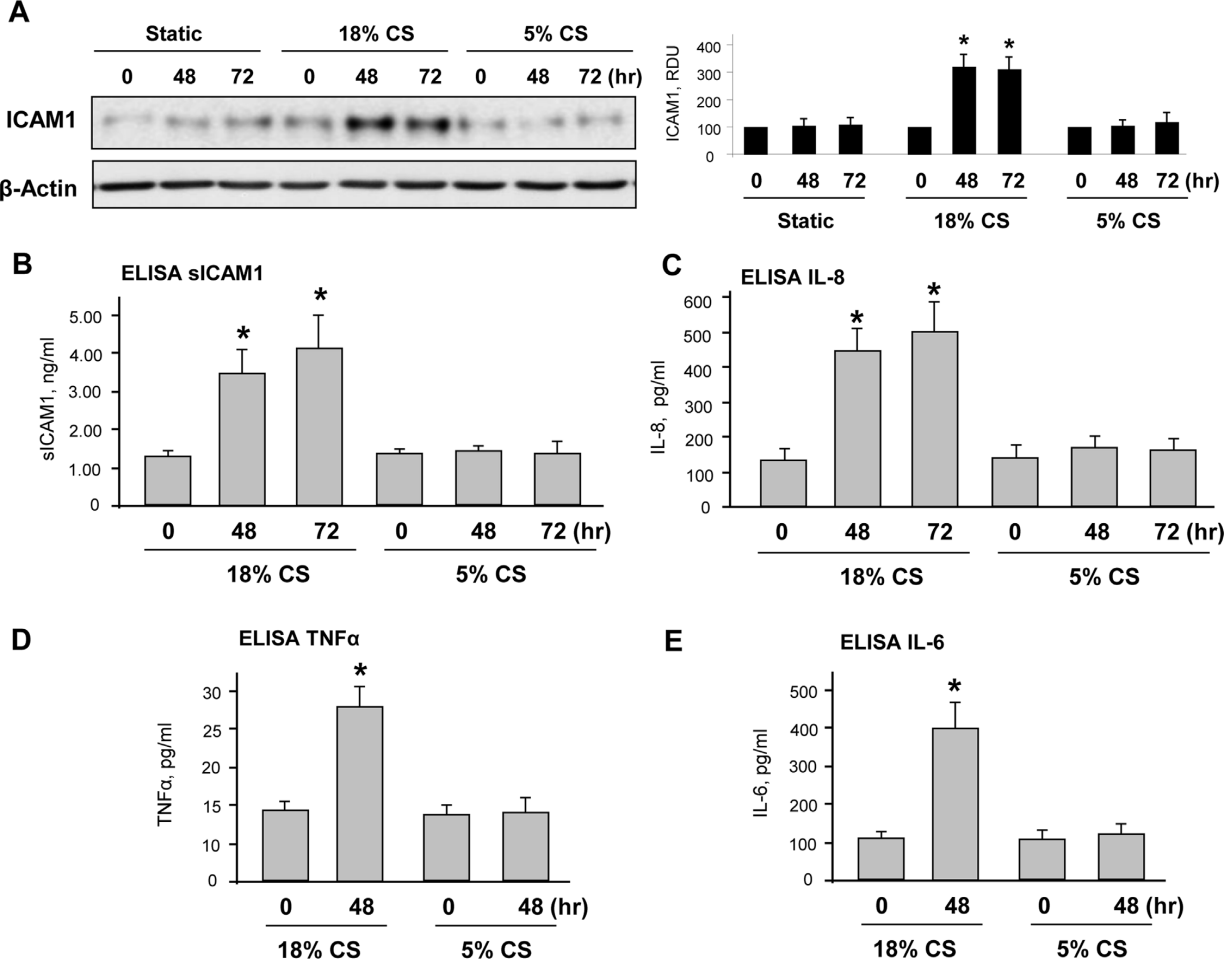
Effects of chronic 5% and 18% CS on pulmonary EC inflammatory activation. HPAEC grown on Flexcell plates were subjected to 18% or 5% CS for 48 or 72 hours. Control cells were left under static conditions. (**A)** ICAM1 expression was detected by western blot with corresponding antibody. β-Actin staining was used as a normalization control; n = 3, *P <0.05 vs. static. Soluble ICAM1 **(B)**, IL-8 **(C)**, TNFα **(D)** and IL-6 **(E)** production in stretch-preconditioned culture media was evaluated by ELISA assay; n = 4, *P<0.05 vs. 5% CS.

Human pulmonary EC grown to confluence on Flexcell plates were exposed to 18% CS, 5% CS or static conditions for 48–72 hours. In contrast to static conditions or physiological 5% CS, chronic preconditioning at 18% CS induced expression of ICAM1 as monitored by western blot analysis of total cell lysates (**Fig 1A**). EC exposure to 18% CS also caused accumulation of soluble ICAM1 and IL-8 in the conditioned medium (**Fig 1B and 1C**), as well as increased levels of TNFα, IL-6 (**Fig 1D and 1E**). Importantly, EC exposure to 5% CS did not affect the basal levels of soluble ICAM1 and three cytokines detected in static EC culture.

### Chronic 18% CS enhanced EC inflammatory response and barrier dysfunction induced by LPS

**Fig 2:**
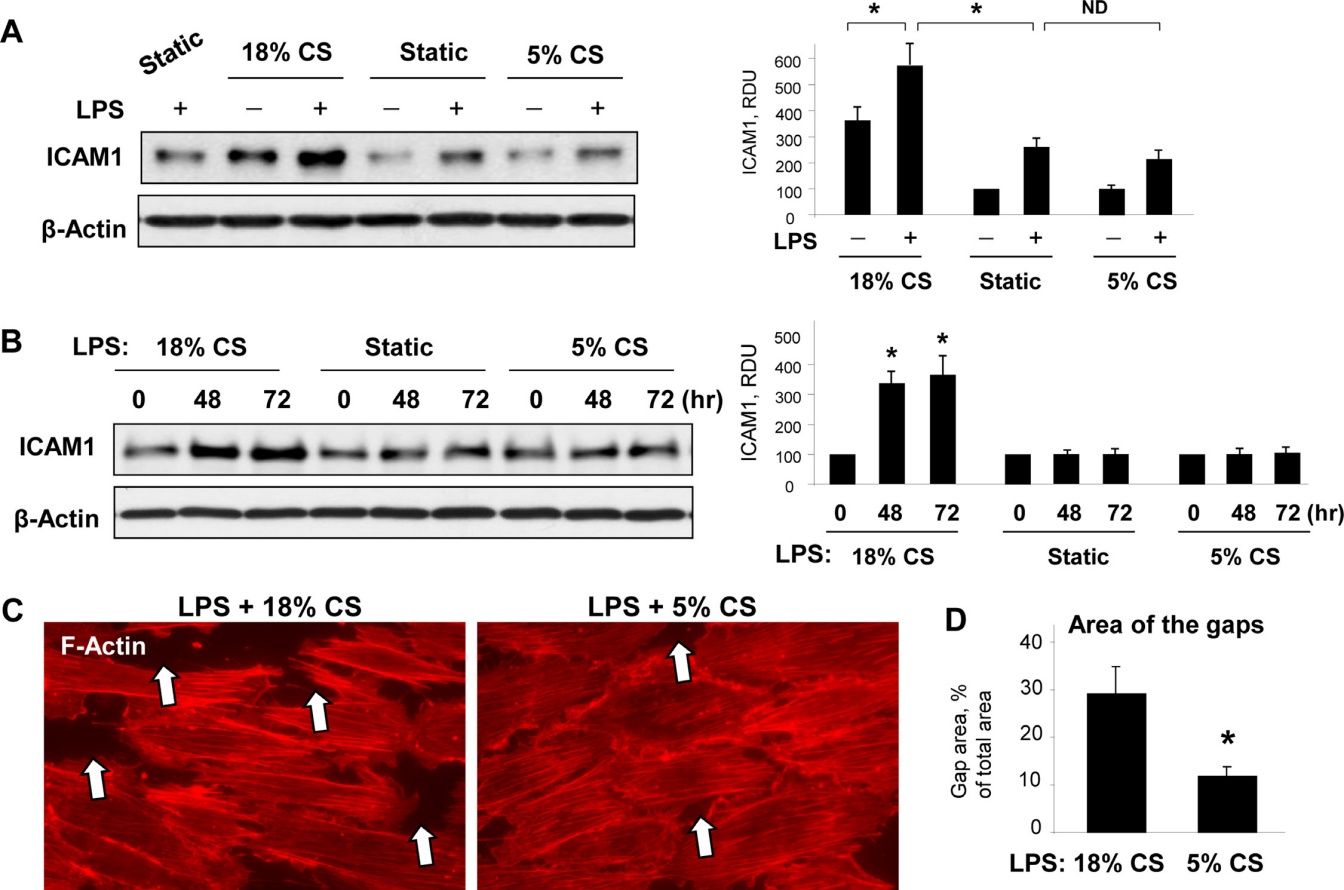
Effect of chronic 18% CS on EC barrier dysfunction induced by LPS. (**A)** HPAEC were subjected to 18% or 5% CS for 72 hours and stimulated with vehicle or LPS (50 ng/ml) in the last 6 hours of stretching. Control cells were left under static conditions. ICAM1 expression was detected by western blot with corresponding antibody. β-Actin staining was used as a normalization control; n = 3, *P <0.05 vs. static. (**B)** ICAM1 expression was analyzed in LPS-treated EC (50 ng/ml, 6 hrs) after stretch preconditioning for 48 or 72 hours. Probing for β-actin served as a normalization control; n = 3, *P <0.05 vs. static. **(C)** EC were subjected to stretch preconditioning at 18% or 5% CS for 72 hours, followed by stimulation with LPS. Cytoskeletal remodeling was examined by immunofluorescence staining for F-actin; paracellular gaps are marked by arrows. **(D)** Quantitative analysis of paracellular gap formation in LPS-stimulated HPAEC subjected to 18% or 5% stretch preconditioning. Data are expressed as mean ± SD; n = 3, *P <0.05 vs. 18% CS.

We further assessed the impact of low and high magnitude CS on EC inflammation induced by LPS. Confluent endothelial cell monolayers were treated with a submaximal dose of LPS (50 ng/ml) added in the last 6 hrs of CS stimulation. LPS-induced activation of ICAM1 expression was significantly enhanced in EC exposed to 72-hr 18% CS compared to static control. Stimulation with 5% CS did not further elevate LPS-induced ICAM1 expression above levels observed in static culture (**Fig 2A**). Similar results were observed in different experimental setting, where EC exposed to 18% CS for 48 or 72 hrs were then placed to static conditions and stimulated with LPS for 6 hrs without CS. LPS-induced ICAM1 expression was augmented in EC preconditioned at 18% CS (**Fig 2B**). Of note, preconditioning at 5% CS did not affect LPS-induced ICAM1 expression.

Effects of chronic exposure to high and low CS on LPS-induced barrier dysfunction were examined in the next experiments. Pulmonary EC conditioned at 5% CS or 18% CS for 72 hrs were treated with LPS during last 6 hrs of CS exposure. 18% CS enhanced LPS-induced gap formation, as compared to LPS-treated EC under 5% CS (**Fig 2C**). The bar graph depicts quantitative analysis of gap formation (**Fig 2D**).

### GEF-H1 mediates EC inflammatory activation induced by chronic high magnitude CS

**Fig 3:**
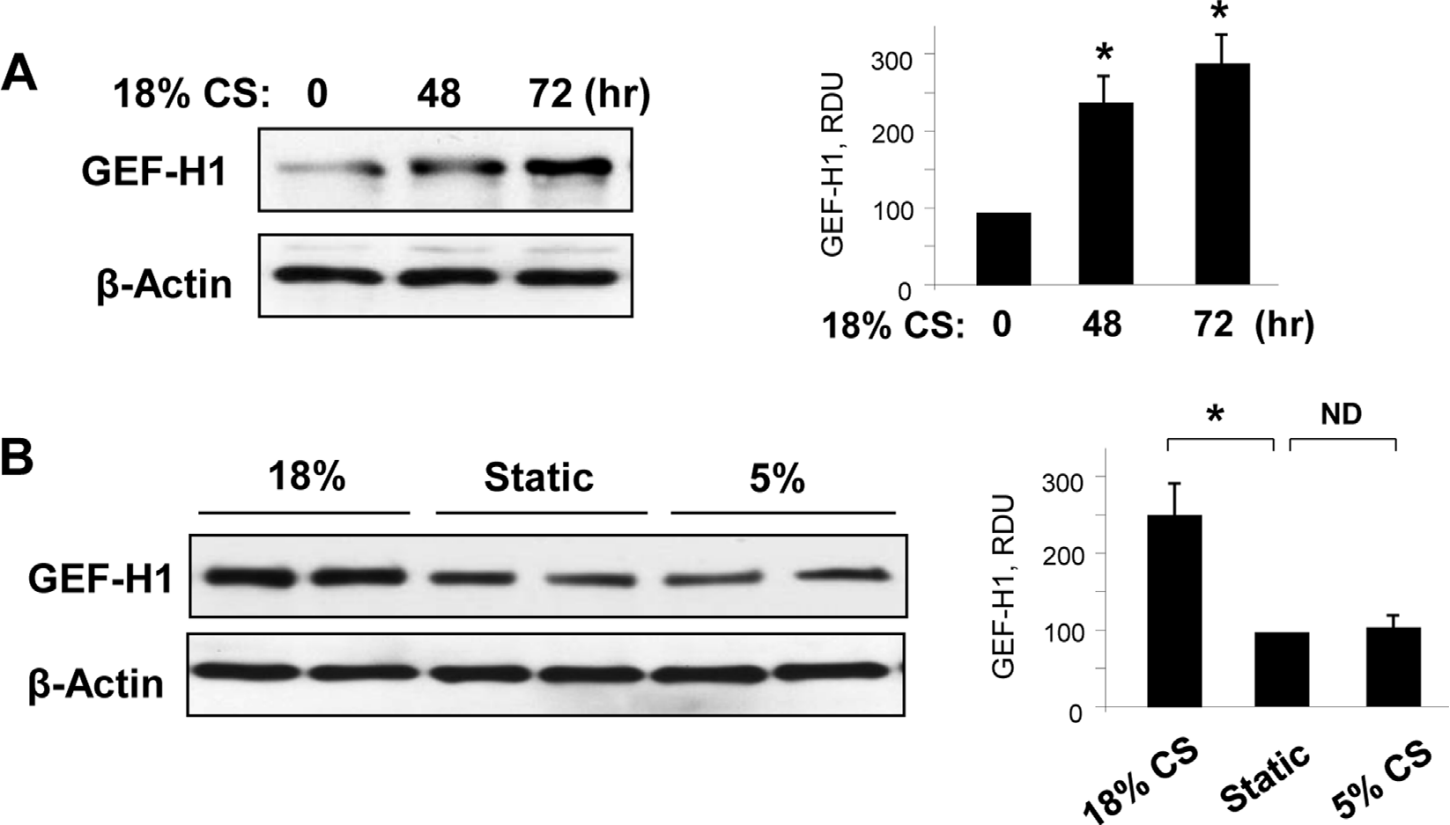
Effect of chronic CS preconditioning on GEF-H1 expression. **(A)** HPAEC were subjected to 18% CS for 48 or 72 hours, or left under static conditions. Stretch-induced time-dependent expression of GEF-H1 was monitored by immunoblotting with corresponding antibodies. Equal protein loading was confirmed by determination of β-actin content in total cell lysates; n = 3, *P <0.05 vs. static. (**B)** HPAEC were subjected to 18% or 5% CS for 72 hours, or left under static conditions. GEF-H1 expression was detected in cell lysates by western blot. β-Actin staining was used as a normalization control; n = 3, *P <0.05 vs. static.

Sensitization of Rho mediated signaling by chronic EC preconditioning at high magnitude CS has been noted in our previous studies [21], but the mechanism remained elusive. Acute activation of Rho-specific activator GEF-H1 by short-term CS is mediated by microtubule disassembly plays a role in rapid activation of barrier-disruptive Rho signaling [17]. We next tested if GEF-H1 can be regulated by chronic CS and involved in EC functional responses to stretch. Chronic 18% CS significantly increased GEF-H1 protein expression observed at 48 hrs and 72 hrs of stimulation (**Fig 3A**). In contrast to 18% CS, chronic exposure to 5% CS did not affect GEF-H1 expression levels, which were comparable to EC under static conditions (**Fig 3B**).

**Fig 4:**
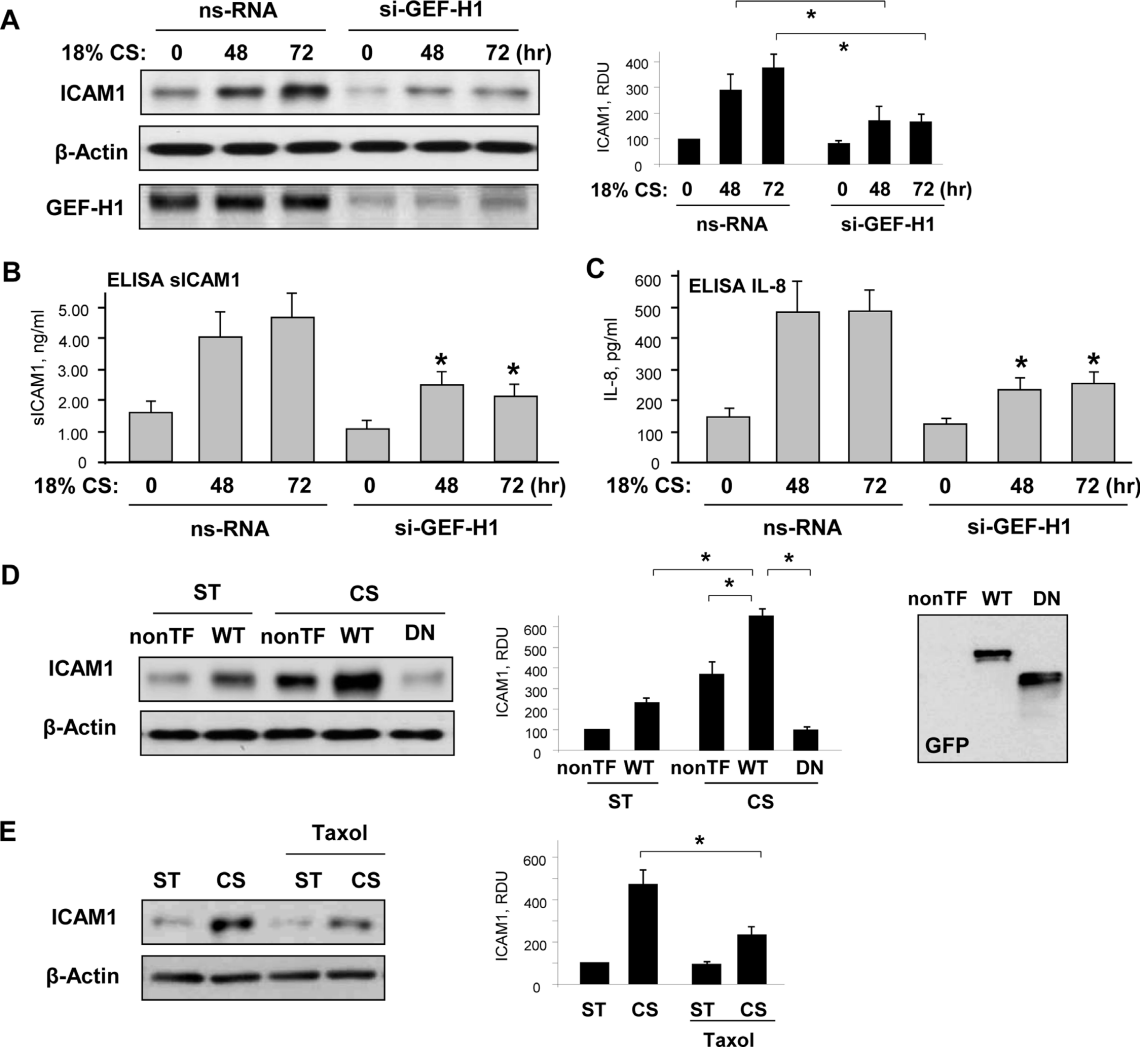
Involvement of GEF-H1 in EC inflammatory activation induced by chronic 18% CS. Cells were transfected with GEF-H1-specific or non-specific siRNA, followed by 18% CS for 48 or 72 hours. (**A)** Effect of GEF-H1 knockdown on CS-induced ICAM1 expression was analyzed in static and stretched cells. Probing for β-actin served as a normalization control. SiRNA-induced protein knockdown was confirmed by western blot; n = 3, *P <0.05 vs. ns-RNA. Effect of GEF-H1 knockdown on CS-induced soluble ICAM1 **(B)** and IL-8 production **(C)** in the culture media was evaluated by ELISA assay; n = 4, *P<0.05 vs. 5% CS. (**D)** Effect of ectopic expression of wild type (GEF-H1-WT) and dominant negative mutant of GEF-H1 (GEF-H1-DN) on CS-induced ICAM1 expression (48 hrs was compared to non-transfected (nonTF) control cells. Probing for β-actin served as a normalization control. Expression of endogenous and recombinant GEF-H1 variants was confirmed by western blot; n = 3, *P <0.05. (**E)** Effect of microtubule stabilization by treatment with taxol (0.5 μM) on CS-induced ICAM1 expression (48 hrs). Probing for β-actin served as a normalization control. Expression of endogenous and recombinant GEF-H1 variants was confirmed by western blot; n = 3, *P <0.05.

The causal role of increased GEF-H1 expression in CS-induced EC inflammatory activation was further tested in experiments with siRNA-induced GEF-H1 knockdown. EC treatment with GEF-H1-specific siRNA abolished 18% CS-induced activation of ICAM1 expression (**Fig 4A**). GEF-H1 knockdown also abolished 18% CS-induced release of sICAM1 and IL-8 into culture medium by CS-stimulated EC (**Fig 4B and 4C**). In contrast, forced expression of GEF-H1 in HPAEC augmented 18% CS-induced upregulation of ICAM1 levels (**Fig 4D**). This effect was not observed in CS-stimulated cells expressing dominant negative GEF-H1 mutant lacking RhoA-specific guanine nucleotide exchange activity. Inset in Fig 4D shows western blot analysis of endogenous and ectopically expressed GEF-H1 variants, and bar graph represents quantitative analysis of ICAM1 protein levels in stimulated cells by densitometry of western blots.

Stabilization of MT by treatment with taxol during EC exposure to chronic CS attenuated CS-induced ICAM1 expression. These data are consistent with reported GEF-H1 sequestration at MT leading to suppression of GEF-H1 activity [16], which in the settings of chronic exposure to pathologic CS attenuated GEF-H1 effects on ICAM1 expression (**Fig 4E**). Taken together, these results show direct involvement of upregulated GEF-H1 in the EC inflammatory response to high magnitude CS.

### Role of GEF-H1 in synergistic effects of chronic high magnitude CS and LPS on EC inflammatory response and barrier dysfunction

**Fig 5:**
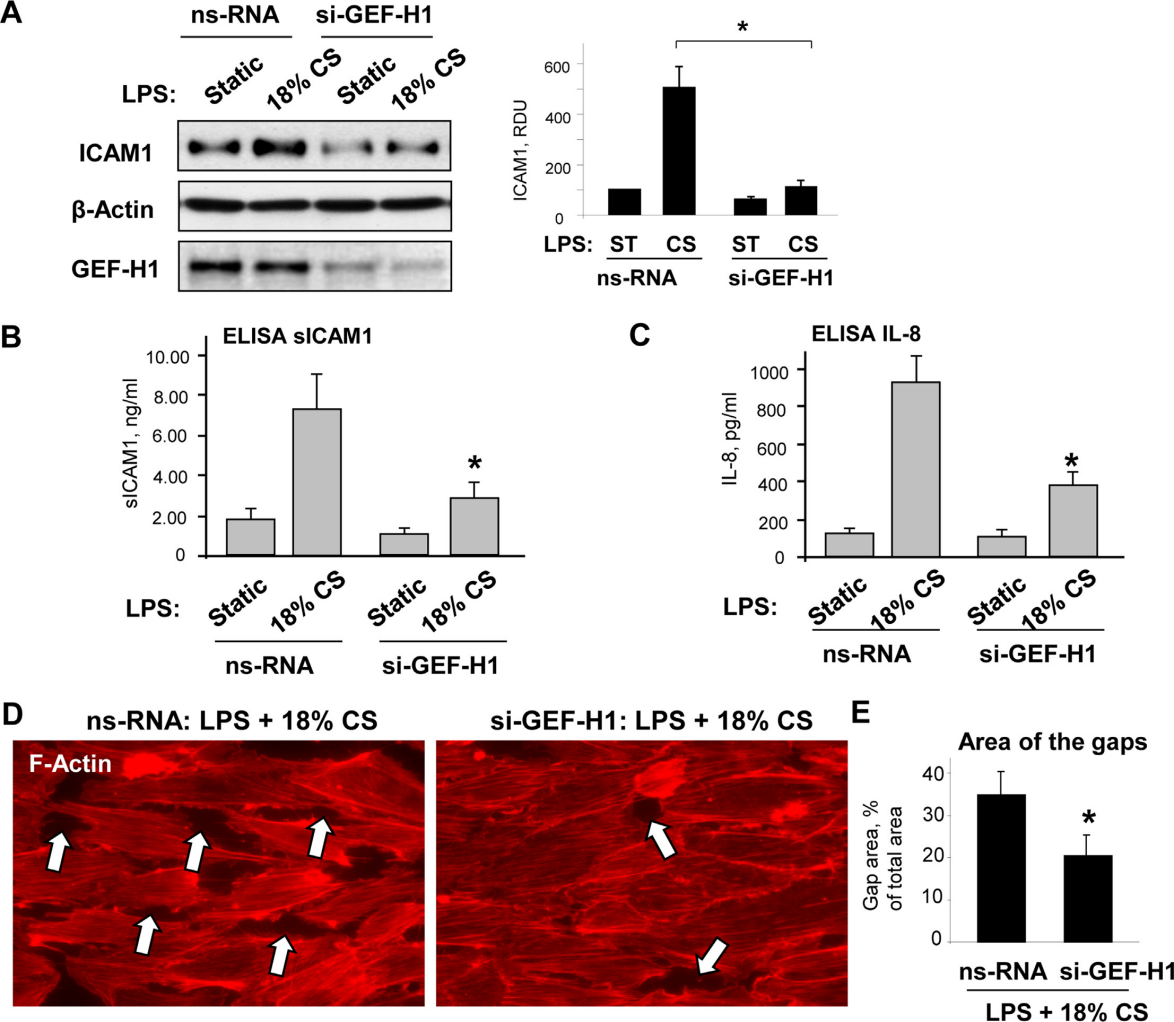
Role of GEF-H1 in synergistic effects of chronic 18% CS- and LPS-induced EC barrier dysfunction. Human pulmonary EC were transfected with GEF-H1-specific or non-specific siRNA. (**A)** Cells were subjected to 18% CS for 72 hours followed by LPS (60 ng/ml, 6 hr) treatment under continuing CS. Control cells were left under static conditions. Effect of GEF-H1 knockdown on LPS/CS-induced ICAM1 expression was examined by western blot. SiRNA-induced GEF-H1 depletion was confirmed by western blot. Probing for β-actin was used as a normalization control; n = 3; *P <0.05 vs. ns-RNA. Effect of GEF-H1 knockdown on soluble ICAM1 **(B)** and IL-8 **(C)** production after combined LPS and 18% CS stimulation was evaluated by ELISA assay. Data are expressed as mean ± SD; n = 4, *P <0.05 vs. ns-RNA. **(D)**Control or GEF-H1-depleted EC were subjected to 18% CS for 72 hours, followed by LPS challenge. Cytoskeletal remodeling was examined by immunofluorescence staining for F-actin; paracellular gaps are marked by arrows. **(E)** Quantitative analysis of 18% CS/LPS-induced paracellular gap formation in control and GEF-H1-depleted EC. Data are expressed as mean ± SD; n = 3, *P <0.05 vs. ns-RNA.

The effect of pathologic CS stimulation on the LPS-induced EC inflammatory response was tested in experiments where pulmonary EC were treated with non-specific or GEF-H1-specific siRNA and exposed to static conditions or 72-hr 18% CS in the presence of LPS. In agreement with the results described above, LPS-induced ICAM1 expression was further augmented by EC exposure to 18% CS, but was dramatically attenuated in cells treated with GEF-H1-specific siRNA (**Fig 5A**). Additional analysis showed that GEF-H1 knockdown abolished synergistic effect of chronic 18% CS exposure on the LPS-induced production of soluble ICAM1 and IL-8 by pulmonary EC (**Fig 5B and 5C**).

We next tested the involvement of GEF-H1 in cytoskeletal remodeling and monolayer integrity of pulmonary EC exposed to chronic high magnitude CS and LPS using a GEF-H1 knockdown approach. HPAEC were transfected with GEF-H1-specific or non-specific siRNA followed by exposure to 18% CS. Actin cytoskeletal remodeling in control and siRNA-treated EC monolayers exposed to 18% CS was analyzed by immunofluorescence staining of F-actin. In contrast to EC transfected with non-specific RNA, GEF-H1 knockdown significantly decreased CS- and LPS-induced increase in stress fibers (**Fig 5D**), and such cytoskeletal relaxation resulted in reduction of paracellular gap formation (**Fig 5E**), reflecting preservation of EC monolayer integrity.

## Discussion

Regional heterogeneity of ventilation in the injured lungs represents a serious problem in defining the optimal regimen of mechanical ventilation of patients with pre-existing respiratory complications in the intensive care unit [2, 32]. As a result, suboptimal mechanical ventilation may cause overinflation of certain lung compartments leading to ventilator-induced lung injury. Pulmonary EC exposed to pathologic mechanical forces relevant to suboptimal mechanical ventilation acquire an activated phenotype and become more susceptible to inflammatory insults [33], but the mechanism of such sensitization remains unclear.

Previous studies by our group characterized acute mechanism of CS-induced activation of GEF-H1 nucleotide exchange activity associated with rapid CS-induced cytoskeletal remodeling, which led to activation of RhoA [17]. In turn, the major focus of this study was comparative analysis of chronic effects of physiological and pathological mechanical stretch on parameters of lung endothelial cell inflammatory activation.

The novel findings of this study are that: a) GEF-H1 expression is upregulated by pathologically relevant 18% CS in pulmonary EC; and b) GEF-H1 increased 18% CS-induced expression of inflammatory molecules ICAM1, VCAM1, IL-8, IL-6 and TNFα and augmented EC inflammatory response to LPS. Importantly, this effect was induced by 18% CS, but not by physiologically relevant 5% CS. The relevance of these CS magnitudes to normal and excessive lung inflation, as well as lung vascular permeability and endothelial responses in animal models of low and high tidal volume mechanical ventilation has been established in earlier studies [21, 26, 34]. However, a serious limitation of existing animal models of mechanical ventilation is inability of prolonged mechanical ventilation which is normally limited to 6–8 hrs. This study shows induction of GEF-H1 expression in human pulmonary endothelial cells exposed to 48-72-hrs of high magnitude CS. The clinical relevance of this study is demonstration of an additional mechanism that may contribute to the lung vascular inflammatory activation caused by chronic ventilation support of critically ill patients (several days of ventilation) which cannot be reproduced in murine models of VILI.

Thus, the CS-induced upregulation of GEF-H1 described in this study may explain exacerbation of agonist-induced Rho signaling and the augmented permeability response in pulmonary EC exposed to pathologic CS. Stimulation of pulmonary EC with high magnitude CS augmented agonist-induced Rho-dependent activation of myosin light chain phosphorylation and actomyosin stress fiber formation, leading to an exacerbated EC contractile response and paracellular gap formation [12, 17]. The key role of GEF-H1 in EC inflammatory activation by chronic exposure to 18% CS was confirmed by experiments with GEF-H1 knockdown, which suppressed these effects. In addition, ectopic expression of GEF-H1 using transient transfection approach augmented ICAM1 expression in cells exposed to 24-hr CS, which was in contrast to non-transfected EC controls exposed to 18% CS. At this time point the increase of endogenous GEF-H1 protein levels is not detectable (data not shown). More importantly, ectopic expression of dominant negative GEF-H1 mutant did not cause activation of ICAM1 expression to the levels observed in EC with overexpressed wild type GEF-H1. These results provide direct evidence of the role of upregulated GEF-H1 expression in CS-induced endothelial inflammatory activation.

Expression of ICAM1, VCAM1 and IL-8 is controlled by a NFκB transcriptional mechanism activated by many pro-inflammatory stimuli. The canonical pathway of NFκB cascade activation by LPS involves activation of TLR-4 receptor leading to recruitment of the adaptor molecules MyD88, IL-1R-associated kinase (IRAK), and TNF receptor-associated factor 6 (TRAF6), and activation of MAP kinases and NFκB pathway [35]. These cascades activate expression of inflammatory cytokines and surface adhesion molecules leading to neutrophil and monocyte adhesion to vascular endothelium, extravasation and activation of tissue inflammation. In addition to this canonical pathway, stimulation of Rho signaling may further enhance NFκB and stress kinase activation leading to elevated expression of inflammatory markers. Rho activation in static cell cultures stimulated transcription of pro-inflammatory genes, while inhibition of Rho signaling reduced expression of TNFα, CXC chemokines, leukocyte infiltration, and endotoxin-induced lung edema [36, 37]. Our previous studies of EC exposed to acute IL-6 and 18% CS stimulation have shown that acute exposure to 18% CS increased activation of p38 stress kinase and NFκB pathways in IL-6-stimulated EC monolayers [33]. This synergistic effect was dependent on Rho activity, inhibited by Y-27632, and resulted in exacerbation of IL-6-induced EC monolayer disruption, lung barrier dysfunction, and inflammation. Thus, although the crosstalk between p38 MAPK, NFκB and Rho signaling becomes increasingly recognized, precise mechanisms and the hierarchy of these interactions require further investigation.

Interestingly, EC exposure to physiologic CS levels neither induced GEF-H1 expression nor affected basal or LPS-induced expression of ICAM1, VCAM1 or IL-8. The nature of different EC sensitivity to low and high CS levels as well as the precise mechanism of magnitude-dependent expression of GEF-H1 still remains to be investigated. For example, partial microtubule destabilization in EC exposed to 18% CS [17] and oxidative stress associated with inflammatory stimulation [19] induced transient GEF-H1 activation. Whether these or other factors contribute to sustained increase in GEF-H1 expression in EC chronically exposed to 18% CS is unknown.

Exacerbation of LPS-induced permeability in 18% CS-preconditioned EC with increased GEF-H1 expression observed in this study may be explained by the higher levels of GEF-H1 activation upon LPS-induced partial disassembly of microtubules. This process leads to release and activation of GEF-H1 [16] and further promotes Rho signaling, EC barrier dysfunction and inflammatory activation [19, 33]. GEF-H1 release from MT cytoskeleton of pulmonary EC in static conditions was correlated with LPS-induced EC permeability, p38 and NFkB activation, ICAM-1 and IL-8 expression, and neutrophil adhesion to activated EC [19].

In conclusion, this study shows that chronic preconditioning of pulmonary EC with high magnitude CS relevant to ventilation at high tidal volume stimulates expression of inflammatory markers by endothelial cells, and this effect is mediated by elevated expression of Rho activator GEF-H1 caused by chronic 18% CS. These findings may explain higher sensitivity of pulmonary endothelium to edemagenic stimuli even after mechanical ventilation was discontinued. Increased ICAM1 and VCAM1 surface expression by CS-activated pulmonary EC and local production of IL-8 may provoke leukocyte chemotaxis to the over-inflated regions of the lung and cause additional damage of these functional (ventilated) lung compartments, leading to exacerbation of lung injury. Augmented inflammatory response to LPS by EC exposed to chronic 18% CS and mediated by increased GEF-H1 expression represents an additional factor complicating the course of ALI/ARDS. The two conditions of mechanical ventilation and septic lung inflammation are often combined in critically ill patients and may lead to severe ALI/ARDS and high mortality. Therefore, pharmacologic targeting of GEF-H1 may be considered as a therapeutic option for more precise treatment of this condition, as opposed to utilization of global Rho inhibitors.
